# Iron-sulfur protein odyssey: exploring their cluster functional versatility and challenging identification

**DOI:** 10.1093/mtomcs/mfae025

**Published:** 2024-05-14

**Authors:** Cindy Vallières, Orane Benoit, Olivier Guittet, Meng-Er Huang, Michel Lepoivre, Marie-Pierre Golinelli-Cohen, Laurence Vernis

**Affiliations:** Université Paris-Saclay, Institut de Chimie des Substances Naturelles, CNRS UPR 2301, Gif-sur-Yvette cedex 91198, France; Université Paris-Saclay, Institut de Chimie des Substances Naturelles, CNRS UPR 2301, Gif-sur-Yvette cedex 91198, France; Université Paris-Saclay, Institut de Chimie des Substances Naturelles, CNRS UPR 2301, Gif-sur-Yvette cedex 91198, France; Université Paris-Saclay, Institut de Chimie des Substances Naturelles, CNRS UPR 2301, Gif-sur-Yvette cedex 91198, France; Université Paris-Saclay, Institut de Chimie des Substances Naturelles, CNRS UPR 2301, Gif-sur-Yvette cedex 91198, France; Université Paris-Saclay, Institut de Chimie des Substances Naturelles, CNRS UPR 2301, Gif-sur-Yvette cedex 91198, France; Université Paris-Saclay, Institut de Chimie des Substances Naturelles, CNRS UPR 2301, Gif-sur-Yvette cedex 91198, France

## Abstract

Iron-sulfur (Fe-S) clusters are an essential and ubiquitous class of protein-bound prosthetic centers that are involved in a broad range of biological processes (e.g. respiration, photosynthesis, DNA replication and repair and gene regulation) performing a wide range of functions including electron transfer, enzyme catalysis, and sensing. In a general manner, Fe-S clusters can gain or lose electrons through redox reactions, and are highly sensitive to oxidation, notably by small molecules such as oxygen and nitric oxide. The [2Fe-2S] and [4Fe-4S] clusters, the most common Fe-S cofactors, are typically coordinated by four amino acid side chains from the protein, usually cysteine thiolates, but other residues (e.g. histidine, aspartic acid) can also be found. While diversity in cluster coordination ensures the functional variety of the Fe-S clusters, the lack of conserved motifs makes new Fe-S protein identification challenging especially when the Fe-S cluster is also shared between two proteins as observed in several dimeric transcriptional regulators and in the mitoribosome. Thanks to the recent development of *in cellulo, in vitro*, and *in silico* approaches, new Fe-S proteins are still regularly identified, highlighting the functional diversity of this class of proteins. In this review, we will present three main functions of the Fe-S clusters and explain the difficulties encountered to identify Fe-S proteins and methods that have been employed to overcome these issues.

## Graphical Abstract

**Graphical Abstract:**
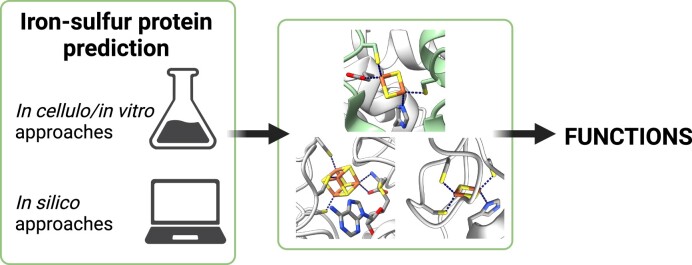
Identification of new iron-sulfur proteins could highlight novel functions for this essential family of proteins.

## Introduction

Iron-sulfur (Fe-S) clusters are among the oldest cofactors on Earth and are essential in the cells. In 1960, Fe-S proteins were detected for the first time by electron paramagnetic resonance (EPR) spectroscopy in mitochondrial membrane; the EPR signal observed upon reduction was different to the EPR signal seen for other metalloproteins.^1^ Two years later, the ferredoxin from *Clostridium pasteurianum* was isolated, and found to harbor non-heme iron and to be involved in electron transport in different low-potential reactions.^2^ Since then, Fe-S proteins have been found in every kingdom of life.

**Fig. 1:**
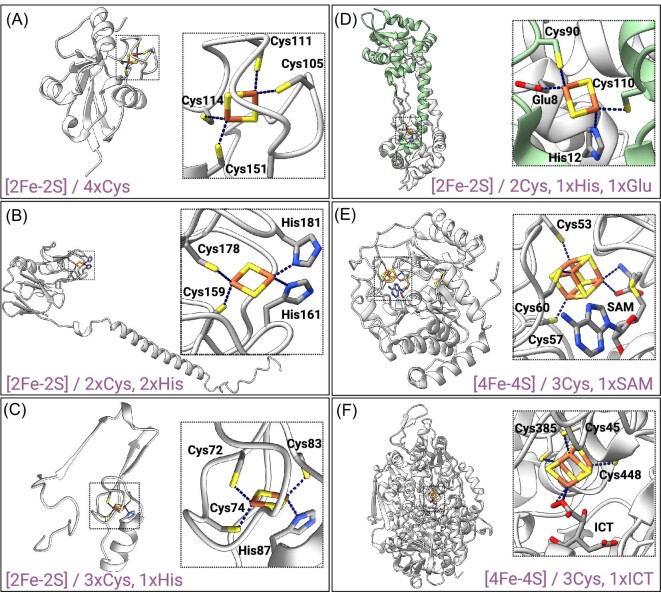
Examples of Fe-S proteins with different Fe-S cluster structure and coordination. (**A**) Human ferredoxin 2 (PDB ID: 2Y5C^11^); [2Fe-2S] coordinated with four Cys residues. (**B**) Rieske subunit of *Saccharomyces cerevisiaebc*_1_ complex (PDB ID: 1KYO^13^); [2Fe-2S] coordinated with two Cys residues and two His residues. (**C**) Human mitoNEET (only one subunit of the homodimer is represented) (PDB ID: 7P0O^15^); [2Fe-2S] coordinated with three Cys residues and one His residue. (**D**) Transcription regulator RsrR from *Escherichia coli* (PDB ID: 6HSD^17^); [2Fe-2S] coordinated with two Cys residues from one subunit of the homodimer (in green) and by His and Glu residues from the other. (**E**) Biotin synthase from *E. coli* (PDB ID: 1R30^19^); [4Fe-4S] cluster coordinated with three Cys residues and an exchangeable S-adenosyl-L-methionine (SAM) molecule (on the right), and [2Fe-2S] coordinated with three Cys and one Arg residues (Fig. 1). (**F**) Aconitase from *Bos taurus* (PDB ID: 1C97^21^); [4Fe-4S] cluster coordinated with three Cys residues and a molecule of isocitrate (ICT).

**Fig. 2:**
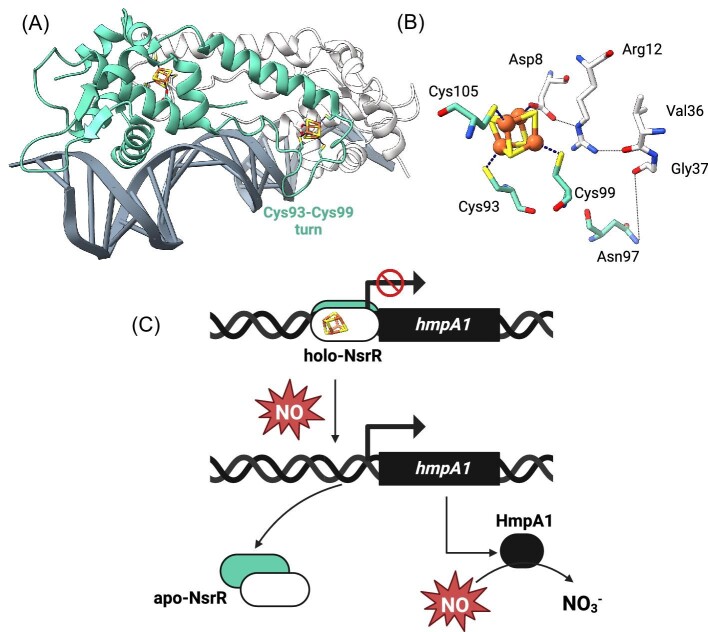
The Fe-S cluster of the NO-sensor NsrR modulates NsrR binding to DNA and subsequent NO stress response. (**A**) The [4Fe-4S]-NsrR from *Streptomyces coelicolor* (in green and white) complexed to 23-bp HmpA1 operator fragment (in grey) (PDB ID: 7B0C^70^). (**B**) The [4Fe-4S] cluster is coordinated with three Cys residues (Cys93, Cys99, Cys105) from one monomer and Asp8 from the other. Disruption of the Arg12-Val36 and as well as the Asn97-Gly37 H-bonds in the absence of cluster results in the displacement of the DNA-recognition helix α3 by about 2.0 Å, which may be enough to prevent the binding of NsrR to DNA. (**C**) Holo-NsrR binds to the DNA upstream of the *hmpA1* gene preventing its transcription. Upon NO stress, NsrR loses its cluster, which leads to the dissociation of NsrR from the DNA and subsequent lift of the transcription repression.

**Fig. 3:**
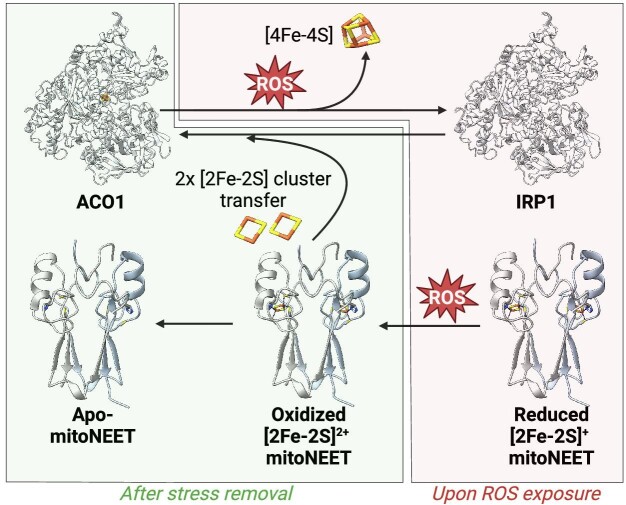
Model involving the Fe-S cluster of human mitoNEET in the repair of the cluster of the cytosolic aconitase after ROS exposure. Under physiological conditions, mitoNEET (PDB ID: 7P0O^15^) harbors a [2Fe-2S]^+^ cluster and aconitase ACO1 (PDB ID: 2B3Y^139^) accommodates a [4Fe-4S] cluster essential for its activity. Upon ROS exposure, mitoNEET [2Fe-2S] cluster is oxidized and aconitase [4Fe-4S] cluster is disassembled revealing the regulatory function of its apo-form termed IRP1 (see Section “Fe-S clusters as Lewis acids: Aconitases and dihydroxyacid dehydratases”). After stress removal, mitoNEET transfers its clusters to IRP1 converting it back to aconitase.

**Fig. 4:**
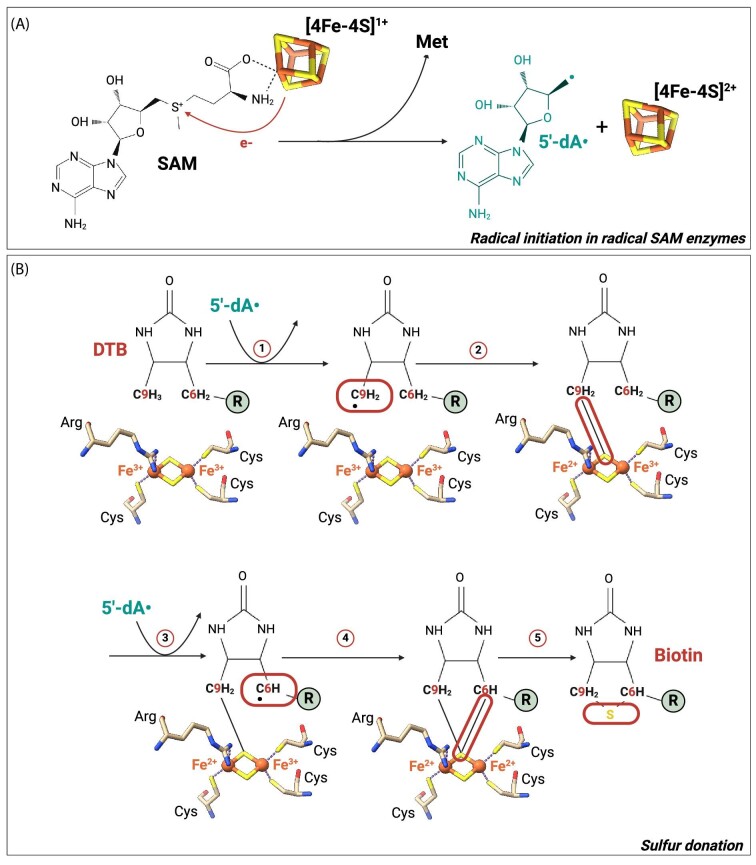
Both clusters of *E. coli* biotin synthase are involved in the conversion of dethiobiotin to biotin. (**A**) *Escherichia coli* biotin synthase BioB (PDB ID: 1R30^19^) contains two cofactors: one [4Fe-4S] and one [2Fe-2S] clusters. The [4Fe-4S] cluster is involved in the reduction and the cleavage of SAM molecules leading to the generation of met residues and 5ʹ-dA• radicals. The later are responsible for the insertion of a sulfur atom, coming from the [2Fe-2S] cluster, between the C6 and C9 carbons of the dethiobiotin (DTB) *via* a radical-based mechanism illustrated in (**B**).

In nature, Fe-S clusters present diverse structures from simple shapes such as [1Fe-0S] clusters, where one iron ion is coordinated by the thiolate group of four cysteines (e.g. rubredoxins^3^), to more complex organizations like [8Fe-7S] clusters found in bacterial nitrogenases.^4^ The most widely observed types are the rhomboid [2Fe-2S] and cubane [4Fe-4S] clusters (Fig. 1).^5,6^ Fe ions connected by inorganic sulfur atoms adopt a distorted tetrahedral coordination. Even though Fe-S clusters can spontaneously assemble under certain conditions, all living cells require a large number of proteins for their biosynthesis, trafficking, and target-specific insertion. These protein machineries termed iron sulfur cluster (ISC), sulfur mobilization (SUF), and nitrogen fixation systems have been extensively described in the literature.^7–9^ Once the cofactors are formed, they are then secured within proteins via the coordination of the Fe ions to the side chain of surrounding amino acids with cysteine being the most common (all Fe-S clusters are coordinated by at least one cysteine). In this review, the term “ligand” refers to the chemical structure that coordinates the Fe-S cluster, i.e. either the side chain of an amino acid within a protein, or a small molecule. The [2Fe-2S] clusters are typically connected to the protein via four cysteines (e.g. ferredoxins, Fig. 1A^10,11^), but ligation involving a histidine residue is also common. Best-known examples include the cluster of the Rieske subunit in respiratory cytochrome *bc*_1_ and photosynthetic cytochrome *b_6_f* complexes coordinated by two cysteines and two histidines,^12,13^ and the clusters of the NEET proteins secured within the protein by three cysteines and one histidine (the term “NEET” comes from the presence in these proteines of the conserved amino acid sequence Asn-Glu-Glu-Thr) (Figs. 1B, C and 3).^14,15^ Occasionally the coordination of [2Fe-2S] and [4Fe-4S] clusters can involve the side chain of other amino acids, such as aspartic acid ([4Fe-4S] cluster of the transcriptional repressor NsrR, Fig. 2B,^16^), glutamic acid ([2Fe-2S] clusters of the transcriptional regulator RsrR, Fig. 1D,^17^), serine (the auxiliary [4Fe-4S] cluster of the lipoyl synthase^18^) or arginine ([2Fe-2S] cluster of the biotin synthase, Fig. 4^19^). Dimeric RsrR contains two [2Fe-2S] clusters, each coordinated by two cysteines from one monomer, and one histidine and one glutamic acid from the other and might be the first examples of Fe-S cofactors bound to a protein by three different types of amino acid side chains (Fig. 1D).^17^ Small molecules can also coordinate the cluster: this is the case of the catalytic [4Fe-4S] clusters of radical S-adenosyl-L-methionine (SAM) enzymes and aconitases, which are ligated within the proteins via three cysteines and a SAM molecule or a water molecule/substrate, respectively (Fig. 1E, F).^19–21^ The monothiol glutaredoxins possess a [2Fe-2S] cluster coordinated by two cysteines residues and two molecules of noncovalently bound glutathione in homo-dimeric complexes.^22^ The type of cluster coordination and more generally the environment surrounding the cofactor is crucial for its function, in such a way that clusters of similar nature but with different coordination and environments might exhibit different functions. Fe-S clusters are usually redox active and typically cycle between two oxidation states. Each Fe atoms of the cofactor can be either in the oxidized state Fe^3+^ (ferric ion), in the reduced state Fe^2+^ (ferrous ion) or be involved in a mixed-valence pair with another Fe atom. Fe-S clusters were primarily known for their role in electron transport, either through small soluble electron carriers like the ferredoxins or in membrane-bound redox enzymatic systems such as photosynthetic and respiratory electron transport chains.^23–27^ They have since been found to be more versatile, playing additional functional tasks including sensing and catalysis in a myriad of fundamental cellular processes such as cellular respiration, gene expression, DNA replication and repair.^28–30^ Nevertheless, while promoting functional diversity, this variety in cluster coordination makes predictions of Fe-S cluster binding and consequently identification of new Fe-S proteins particularly difficult. Moreover, Fe-S proteins are often mistaken for other metalloproteins and have thus been mis-annotated in databases in numerous occasions (e.g. Maio *et al.*,^31,32^ Van *et al.*^33^). Another hurdle for the identification of Fe-S proteins is the sensitivity of the Fe-S clusters to oxygen: Fe-S protein purification in presence of oxygen can lead to cluster disassembly especially when the cofactor is exposed and subsequent mis-annotation. Fortunately, current research efforts and development of proteomics approaches^34^ and tools such as Deep Mind's AlphaFold2 software^35,36^ are greatly contributing in identifying new Fe-S proteins.

We will first review three important functions of the [2Fe-2S] and [4Fe-4S] clusters (i.e. electron transport, sensing, and catalysis). Of note, this review does not aim at cataloguing all known Fe-S proteins and their functions but will focus on a subset of this family of proteins to illustrate their importance and complexity. We will explain further why identifying such proteins remains a problem and what methods have been recently exploited to overcome this.

## Biological functions of the [2Fe-2S] and [4Fe-4S] clusters

Fe-S clusters have been found so far to be versatile protein prosthetic groups that perform four main functions in the cells: electron transport, catalysis, sulfur donation, and sensing. In electron transport, Fe-S clusters employ redox cycling of the Fe atoms to transport electrons to an acceptor. The reduction of the acceptor results in either the direct functional activation of the protein or further electron transfer to other acceptor(s) (e.g. Read *et al.*,^23^ Schulz *et al.*^26^). Their range of redox potentials and their sensitivity to an oxidative environment make them good candidates for sensing environmental changes. Upon changes in iron, oxygen, or reactive oxygen or nitrogen species (ROS or RNS) cellular levels, the Fe-S clusters can be altered (i.e. change in redox state of the cluster, cluster interconversion or cluster occupancy) leading for example to conformational change of the Fe-S proteins that can affect their binding to diverse macromolecules.^37,38^ Fe-S clusters function also as cofactors in enzyme catalysis for instance to perform Lewis acid reactions or to assist in the generation of 5ʹ-deoxyadenosyl radicals (5ʹ-dA•), which initiate various radical reactions.^39,40^ Another function is sulfur donation, where a sulfur atom from the Fe-S cluster is inserted into cofactors such as biotin and lipoic acid.^41^ In few instances, a functional role of an Fe-S cluster is unknown or debated. In this section, we will document the diverse Fe-S cluster functions through some of the best-known and -characterized Fe-S proteins.

### Fe-S proteins involved in electron transport

#### The ferredoxins

Ferredoxins were the first discovered Fe-S proteins.^2^ They are small soluble (<200 residues) and highly acidic proteins. [2Fe-2S] ferredoxins have generally low reduction potentials that range between −500 and −150 mV.^5,42^ Their clusters reside near the protein surface but are protected from degradation by surrounding hydrophobic residues.^43^ The [2Fe-2S] ferredoxins can be grouped into three classes: plant-type, adrenodoxin, and thioredoxin-like ferredoxins, all three groups performing electron transport.^5,24–26,44^ Plant-type ferredoxins function primarily as electron transporters in photosynthesis and are also involved in a variety of other functions such as sulfur and nitrogen assimilation and chlorophyll biosynthesis.^25^ Adrenodoxin-type ferredoxins act as electron transporters in cofactor synthesis (i.e. Fe-S clusters, haem *a*, ubiquinone, lipoic acid) and steroid conversion.^26^ The function of thioredoxin-like ferredoxins remains elusive. Similar to other [2Fe-2S] ferredoxins, thioredoxin-like ferredoxins participate in electron transport, but a regulatory function has also been proposed for this class of proteins.^45,46^ They may act as a thiol-based molecular switch by modulating disulfide bond formation in target proteins as described recently for the yeast *Saccharomyces cerevisiae* [2Fe-2S] protein Aim32.^44,47^ Interactions between the [2Fe-2S] ferredoxins and their redox partners involve electrostatic interactions of charged surfaces that bring the proteins in proximity. Water repulsion at the hydrophobic interface and subsequent conformational rearrangements of the proteins facilitate the interaction and electron transfer.^48^ Contact surface between both partners is not completely complementary ensuring the separation of oxidized ferredoxin and initiation of a new cycle. The plant-type and adrenodoxin-type ferredoxins coordinate their cluster via four cysteines in similar conserved motifs, Cys-X_4_-Cys-X_2_-Cys-X_29_-C and Cys-X_5_-Cys-His-X-Cys-X_35-37_-Cys, respectively (Fig. 1A). The overall fold and structure of both classes are also very similar despite low sequence similarity. In contrast, coordinating cysteine residues in thioredoxin-like ferredoxins are much further apart in the sequence than the two other ferredoxin types (Cys-X_10-12_-Cys-X_29-34_-Cys-X_3_-Cys) and their clusters is also more at the surface.^5^ Proteins from this class function as a dimer. Recently in yeast, two proteins, mitochondrial Aim32 (already mentioned) and its cytosolic counterpart Apd1, presenting a thioredoxin-like ferredoxin domain, were found to coordinate a [2Fe-2S] cluster via two cysteines ligating the ferric ion and two histidines ligating the reducible iron ion (C-X_8_-C-X_24-75_-H-X-G-G-H motif).^49^ The histidine ligands in these proteins enable proton-coupled electron transfer, which so far was only encountered in Rieske and NEET proteins described later in this review.^50–53^ Ferredoxins can also harbor one or two [4Fe-4S] clusters and more exceptionally one [3Fe-4S] cluster. The [4Fe-4S] ferredoxins are mostly found in anaerobic bacteria and present a cofactor mainly coordinated by four cysteines present in a Cys-X_2_-Cys-X_2_-Cys-X_n_-Cys motif. In *Pyrococcus furiosus*, the cluster is however coordinated by three cysteines and one aspartic acid residue.^5,54^ Like [2Fe-2S] ferredoxins and other proteins involved in electron transport, [4Fe-4S] ferredoxins have relatively low reduction potentials (from −650 to −250 mV) except for a subset of [4Fe-4S] ferredoxins called HiPIPs for high potential iron-sulfur proteins (from +100 to +400 mV) that are found in photosynthetic bacteria, where they function in anaerobic electron transport chains. The cofactor in HiPIPs is in a hydrophobic pocket, allowing for the high potential [4Fe-4S]^2+/3+^ redox couple, and is coordinated by four cysteines present in a Cys-X_2_-Cys-X_8-16_-Cys-X_10-13_-Gly-Trp or Tyr-Cys motif.^5^ The wide range of reduction potentials enables the proteins from this family to serve as redox partners to a variety of proteins in a large number of biological reactions.

#### The Rieske(-type) proteins

Another class of [2Fe-2S] proteins involved in electron transport are proteins containing Rieske centers, i.e. Rieske proteins that are catalytic subunits of the cytochrome *bc*_1_ and cytochrome b_6_f complexes, and Rieske-type proteins that encompass a group of aromatic-ring-hydroxylating dioxygenases and arsenite oxidases.^5,27,50^ Rieske and Rieske-type proteins share the conserved coordination motif Cys-X-His-X_15-47_-Cys-X_2_-His (Fig. 1B).^5,12^ Like Aim32 and Adp1 described earlier, one iron ion is coordinated by two cysteines while the other is coordinated by two histidines. Rieske proteins contain two extra cysteines in the motif that do not coordinate the cluster but are still needed for cluster stability by potentially forming a disulfide bridge.^12,55^ The absence of these two cysteines in Rieske-type proteins could be explained by the position of the Fe-S cluster; the cofactor being buried and consequently being less solvent-exposed, extra structural stabilization may not be required.^5^ The reduction potentials of Rieske centers, ranging from −150 to +400 mV, are higher than those observed for [2Fe-2S] ferredoxins.^5^ This can notably be explained by the difference in electronegativity between the histidine and cysteine ligands. The histidine ligands are functionally relevant since they enable coupling of electron and proton transfer during quinol oxidation in respiratory and photosynthetic electron transfer chains.^50^ The [2Fe-2S]^2+^ cluster in the Rieske subunit accepts an electron and a proton upon quinol oxidation leading to its reduction to the mixed-valence state and the protonation of the histidine ligand increasing reduction potential. The cluster then transfers an electron to cytochrome *c* or *f* and the proton to a nearby base. The Rieske protein is not the only Fe-S subunit of the photosynthetic and respiratory complexes. For instance, in the human mitochondrial respiratory chain, six [4Fe-4S] and two [2Fe-2S] clusters are found in the NADH dehydrogenase, and a [4Fe-4S], [3Fe-4S], and [2Fe-2S] clusters are present in the succinate dehydrogenase. Fe-S clusters within these complexes form an electron transfer chain, ensuring electrons transport from the electron carriers NADH and FADH_2_ to ubiquinone, thanks to appropriate redox potentials. The electron transfer in the NADH dehydrogenase is coupled to the translocation of four protons across the mitochondrial inner membrane. In addition to its respiratory role, the succinate dehydrogenase also plays a role in pathways that control metabolism and cell fate.^56^

#### Hydrogenases and nitrogenases

Hydrogenases, found in archaea, bacteria and some eukaryotes catalyze the reversible oxidation of hydrogen. They can be classified into three subgroups based on their metal content and catalytic sites, i.e. [Fe-Fe], [Ni-Fe] and [Fe] hydrogenases. [Fe-Fe] and [Ni-Fe] hydrogenases possess a complex catalytic H-cluster (a [4Fe-4S] linked to a [2Fe] via a bridging cysteine residue), and several [2Fe-2S] and [4Fe-4S] clusters that serve as an electron-transport chain (the number of Fe-S clusters is organism dependent).^57^ It is worth noting that the [Fe-Fe] hydrogenase of the green algae *Chlamydomonas reinhardtii* has only the H-cluster. These clusters are deeply buried within the protein. Nitrogenases are present in a specific group of microorganisms termed diazotrophs and are responsible for the irreversible reduction of nitrogen to ammonia. Similar to the hydrogenases, nitrogenases possess several metallocofactors that are critical for their functions. The well-characterized molybdenum-dependent nitrogenases consist of two proteins: the reductase and the catalytic component. They possess two complex clusters termed M-cluster ([Mo Fe7 S9 C-homocitrate]) and P-cluster ([8Fe-7S]), and a [4Fe-4S] cluster coordinated by four cysteine residues.^57^ During catalysis, electrons received from a ferredoxin or flavodoxin navigate from ATP molecules to the [4Fe-4S] cluster, to the P-cluster, and finally to the M-cluster where the reduction of nitrogen to ammonia takes place.

### Fe-S proteins as sensors

Fe-S clusters can sense notably oxygen, NO, unbalanced redox state of the cells, and low levels of iron and Fe-S clusters. These signaling molecules can alter the redox state (e.g. NEET proteins, RsrR, DNA glycosylases) or induce the degradation (e.g. IscR and NsrR) or conversion (e.g. fumarate nitrate regulator FNR) of the clusters. This characteristic is exploited by the cells for example to control gene expression or to repair macromolecules.

#### Regulators of gene expression: the members of the Rrf2 family and the FNR as examples

One of the best characterized Fe-S sensors are the bacterial FNR and the members of the Rrf2 family of dimeric bacterial transcription factors such as the redox balance-sensing [2Fe-2S]-RsrR, the Fe-S-sensing [2Fe-2S]-IscR, and the NO/RNS-sensing [4Fe-4S]-NsrR. They are all essential for many bacteria. As they are absent in humans, they represent potential targets for the design of novel antibiotics.

*Streptomyces venezuela* RsrR contains one [2Fe-2S] cluster per monomer that senses the redox status within the bacteria. RsrR by binding to promoter DNA sequences regulates several genes associated with the synthesis of mycothiol, the equivalent of glutathione in *Actinobacteria*, and the expression of *nmrA*, which encodes a NAD(P)-dependent transcriptional regulator that senses the cellular redox status via the NAD(P)^+^-to-NAD(P)H concentration ratio.^58,59^  *Sv*RsrR cofactor is asymmetrically coordinated by two cysteines from one monomer and one histidine and one glutamic acid residues from the other, and switches between reduced (+1) and oxidized (+2) states, with only the latter binding DNA with high affinity (Fig. 1D).^17^ The unusual coordination might be important to modulate the cluster's reduction potential as substitution of the histidine/glutamic acid by cysteines impaired redox cycling and subsequently abolished RsrR binding to DNA. The reduction of the cluster triggers the protonation of the histidine ligand and the rotation of a nearby tryptophan (Trp9) from “exposed” to “buried” state, causing conformational changes in the DNA-binding helix-turn-helix region that led to DNA dissociation.^17,60^

Iron starvation leads to a decreased expression of the *isc* operon and an elevation of the expression of the *suf* operon. The small RNA RyhB was proposed to be involved in this transition by base-pairing to *iscRSUA* polycistronic mRNA, inducing the degradation of the 3ʹ region of the mRNA containing *iscSUA* and encoding the Fe-S synthesis machinery. Opposite, the 5ʹ region encoding iscR remains stable.^61^ IscR regulates the expression of the ISC and SUF biogenesis pathway and other 40 genes in *Escherichia coli*.^62–65^ The protein contains a [2Fe-2S] cluster that can sense, oxygen, ROS and possibly RNS, in addition to Fe-S cluster status in the cell. The cluster is coordinated by three cysteines and one histidine. The latter might increase cluster sensitivity to signaling molecules and cluster lability.^66^ Two types of IscR-binding sites exist in IscR-regulated promoters: type 1 found upstream of genes encoding for IscR and proteins of the ISC biogenesis pathway (e.g. *isc* operon) and type 2 found for instance upstream of the *suf* operon, which encodes for proteins that mediate Fe-S cluster assembly under oxidative stress and iron limitation conditions. Holo-IscR (as opposed to apo-IscR that does not harbor a cluster) represses the *isc* operon while apo-IscR activates the *suf* operon.^64,67^ Iron limitation and oxidative stress drive an increase in Fe-S cluster cellular demand. Under these conditions, there is a competition between newly synthesized apo-IscR and other Fe-S cluster recipient proteins for clusters produced by the ISC machinery. Apo-IscR accumulates and activates the expression of the *suf* operon.^64,65^ On the opposite, apo-IscR cannot bind the promoter region of the *isc* operon, and thus cannot prevent the binding of the transcription machinery and subsequent transcription of this operon.^63,66^ The amount of Fe-S cluster generated is thus at its highest level. Other members of the Rrf2 family that can sense iron limitation include RisR and RirA that are both harboring a [4Fe-4S] cluster.^68,69^  *Myxococcus xanthus* holo-RisR is a repressor of both the *isc* and *suf* operons, in contrast to holo-IscR that represses only the *isc* operon. The cluster is coordinated by three cysteines and an unknown fourth ligand.^68^ RirA found in *Alphaproteobacteria* possesses an [4Fe-4S] cluster that enables RirA binding to promoter sequences, thereby causing the repression of cellular iron uptake. Under iron-depleted condition, one of the iron atoms dissociates which leads to RirA cluster disassembly and loss of DNA-binding affinity. RirA cluster was proposed to be coordinated by three cysteines and a non-protein ligand such as water; this would be functionally relevant as the introduction of a fourth cysteine ligand significantly reduced the lability of the fourth iron atom and the ability of the [4Fe-4S] to disassemble under iron depletion.^69^ It could be hypothesized that RisR fourth iron may also be ligated by a non-protein ligand, as both clusters sense low-iron concentration.

Like IscR, RisR, and RirA, NsrR oscillates between its apo- and holo-form. NsrR acts as a regulator of NO-induced stress response in many bacterial species. NsrR controls more than 60 genes in *E. coli*, and less than 20 in *Streptomyces coelicolor* including the *hmp* gene which encodes a NO-detoxifying flavohemoglobin (Fig. 2C). The [4Fe-4S] cluster from *Sc*Nsr Ris ligated by three residues from one monomer (i.e. 3 cysteines) and an aspartic acid from the other (Fig. 2A, B).^16,70^ In presence of the cluster, the sequence contained between both cysteine ligands Cys93 and Cys99 forms a well-defined turn properly oriented to interact with the DNA backbone (Fig. 2A). Asn97 from this turn as well as Arg12, which forms a salt bridge with the aspartic acid ligand Asp8 connecting the cluster to a turn preceding the DNA-binding helix α3, are critical to modulate the orientation of this helix at the major groove of the nucleic acid (Fig. 2B).^16^ NO causes the breaking of both inter-monomer Asp8-[4Fe-4S] bonds initiating cluster degradation and structural changes that prevent DNA binding.^16^ Carboxylate are more labile than thiolate ligands, and presence of such ligands is important for the protein to maintain controlled, concerted nitrosylation across both clusters.^71^

Bacterial FNR controls the switch between aerobic and anaerobic metabolism by regulating the transcription of hundreds of genes in response to oxygen levels in bacteria. However, instead of using cluster occupancy as a sensing mechanism, the Fe-S cluster of the FNR coordinated by four cysteines cycles between a [4Fe-4S] cluster in anaerobic conditions and a [2Fe-2S] cluster in the presence of oxygen. Oxygen induces the oxidation of the cysteines ligating the [4Fe-4S] cluster leading to the formation of a semi-stable cysteine persulfide-ligated [2Fe-2S] cluster. This (reversible) interconversion results in a dimer-to-monomer transition and loss the ability of FNR to bind DNA.^72–74^

#### Repair proteins: the NEET protein mitoNEET and DNA glycosylases

NEET proteins form a class of [2Fe-2S] proteins, present in all the kingdoms of life. In mammals, this family is composed of three members: mitoNEET (CISD1), CISD2 (NAF-1, miner1) and CISD3 (miner2, MiNT) while plants only contain one member known as AtNEET in *Arabidopsis thaliana*.^75,76^ MitoNEET and CISD2 are bound to the outer mitochondrial and endoplasmic reticulum membranes, respectively, with the main part of the proteins, which contains the cofactors, present in the cytosol, while CISD3 is reported to be localized in the mitochondrial matrix.^77^ NEET proteins are characterized by the presence of at least one highly conserved CDGSH iron sulfur domain (CISD) containing the consensus sequence [C-X-C-X_2_-(S/T)-X_3_-P-X-C-D-G-(S/A/T)-H]. These proteins are an example of mis-annotated Fe-S proteins in databases. They were indeed thought to bind a zinc molecule through the cysteine and histidine residues of the CISD conserved domain.^77^ MitoNEET is a 108 amino acid protein first discovered in 2004 as a target for pioglitazone used to treat type 2 diabetes.^78^ When purified from *E. coli*, the protein was of brownish/red color suggesting the presence of iron. Metal content analysis confirmed the presence of iron in the protein and excess of zinc did not favor zinc insertion.^77^ The optical spectrum of the purified mitoNEET exhibited two peaks at 458 and 530 nm characteristics of the presence of a [2Fe-2S] cluster.^79^ To identify the residues involved in the coordination of the cofactor, the residues that were conserved in the three NEET proteins, i.e. Cys72, Cys74, Cys83, His87, and Asp84 were systematically mutated by a serine for the cysteine residues and glutamine and asparagine for histidine and aspartic acid residues, respectively. The effects of the mutations were measured by UV–visible (UV–Vis) absorption spectroscopy and a loss of signal was observed in all the mutants except in the mutant D84N suggesting a coordination of the [2Fe-2S] by the three cysteines and the histidine (Fig. 1C).^79^ Although their physiological and molecular roles are still debated, NEET proteins seem involved in several essential cellular pathways including regulation of autophagy, ferroptosis, iron, calcium, and ROS homeostasis and participate in the cellular adaptative response to redox perturbations.^80–82^  *In vitro*, mammalian NEET proteins can transfer their cluster to a recipient protein if the cluster is oxidized but not if reduced (Fig. 3).^83–86^ It was proposed that mitoNEET can repair the Fe-S cluster of cytosolic aconitase (ACO1) when damaged by an oxidative/nitrosative stress using its cluster transfer ability (Fig. 3).^85^ However, how the two [2Fe-2S] clusters would be reductively coupled to generate the [4Fe-4S] of ACO1 is not known. NEET proteins act as redox switch proteins: the oxidative state of their Fe-S cluster controls their activity in response to redox signals (Fig. 3).^83,84^ Recent studies showed that oxidized mitoNEET can also gate the voltage-dependent anion channel 1 (VDAC1).^10^ This sensing function can participate to the protection of the cells from environmental changes by enhancing different mechanisms involving Fe-S cluster or electron transfer.^84^ Dysregulation of the NEET protein level is observed in several severe pathologies including cancer and neurodegenerative diseases.^80,87^ Targeting NEET proteins to disturb these regulations, in order to treat pathologies in which NEET proteins play an important role, has recently emerged as a potential therapeutic strategy.^88,89^

Fe-S clusters are known to be essential components of prokaryotic and eukaryotic nucleic acid processing machineries including glycosylases, primases, helicases, nucleases, and tRNA-modifying enzymes.^29,90^ The [4Fe-4S] clusters of prokaryotic and eukaryotic DNA glycosylases involved in DNA repair (e.g. endonuclease III and MutY) and coordinated by four cysteines in a Cys-X_6_-Cys-X_2_-Cys-X_5_-Cys motif have been long thought to only play a structural role.^91,92^ However, recent studies have suggested that they might also display a sensing function. A repair enzyme with a [4Fe-4S]^2+^ cluster may bind DNA, be oxidized, and subsequently release an electron that will use DNA charge transfer to travel along the DNA chain until it meets another DNA-bound repair enzyme. It may then reduce its [4Fe-4S] cluster, leading to the subsequent release of this repair enzyme from the DNA. This released protein can scan another DNA region. In the event where a damage is present between both DNA repair enzymes, the DNA charge transfer may be disrupted and the second enzyme will remain bound, proceeding to scan along the DNA to find and repair this damage.^93–95^ This “scanning” function in DNA repair is however still disputed.

### Fe-S proteins in (non-)redox catalysis

Several enzymes with [2Fe-2S] and [4Fe-4S] clusters function in either non-redox or redox catalysis.

#### Fe-S clusters as Lewis acids: aconitases and dihydroxyacid dehydratases

The [4Fe-4S] aconitases catalyze the stereo-specific isomerization of citrate to isocitrate with an intermediate metabolite, *cis*-aconitate, in the tricarboxylic acid (TCA) cycle that provides electrons to the electron transport chain leading to the generation of ATP. The TCA cycle is also crucial for the biosynthesis of various metabolites including citrate, isocitrate, succinate, fumarate, and oxaloacetate. Only three of the four Fe ions of their clusters have cysteine ligands; the labile Fe ion is bound to oxygen atoms from either water or substrates to be dehydrated and acts as a Lewis acid to activate the hydroxyl group of the citrate substrate (Fig. 1E). Upon oxidation, the enzyme loses the fourth iron to form a [3Fe-4S] cluster, which is catalytically inactive (Fig. 3).^40^ Humans possess mitochondrial and cytosolic aconitases. The cytosolic protein is a moonlighting enzyme; the holo-protein ACO1 functions as an aconitase while the apo-protein termed IRP1 is an iron response protein involved in the regulation of iron homeostasis. The apo-protein binds to iron-responsive elements present in the 5ʹ or 3ʹ UTR of target mRNA to promote iron uptake and reduce iron storage and utilization.^96^  *Escherichia coli* (and other bacteria) also possesses two aconitase isozymes, AcnA and AcnB, that are both involved in the TCA cycle. AcnB is considered to be the major aconitase in the TCA cycle, while AcnA is specifically expressed under stress conditions, exhibiting a more stable [4Fe-4S] cluster.^97^ Apo-AcnA and -AcnB bind to the 3ʹ-UTR of *acnA* and *acnB* mRNAs to stabilize them, in a positive control loop.^97,98^

Dihydroxyacid dehydratases (DHAD) are homodimeric Fe-S enzymes of 60–70 kDa per monomer that catalyze the fourth step in the biosynthesis of isoleucine and valine namely the dehydratation of 2,3-dihydroxy-isovaleic acid into α-ketoisovaleric acid.^99^ While *E. coli* DHAD contains an oxygen-labile [4Fe-4S] cluster, spinach DHAD as well as *S. cerevisiae, A. thaliana*, and *Mycobacterium tuberculosis* DHAD harbor an oxygen-resistant [2Fe-2S] cluster.^100–103^ The Fe-S cluster of these enzymes is also coordinated by three cysteines and one non-cysteinyl ligand and acts as a Lewis acid to activate the 3-hydroxy group of the substrate during the catalytic cycle.^102,104^

#### Fe-S clusters as initiators of radical chain reactions: the radical SAM superfamily

Fe-S cluster can be part of redox catalysis. One of the best-characterized examples is the radical SAM superfamily. This is the largest superfamily of metal-containing enzymes, with over 100 000 members among which only a few have been fully characterized. These enzymes catalyze various radical reactions using a [4Fe-4S]^+^ cluster to perform a reductive cleavage of a SAM molecule to L-methionine and 5ʹ-dA• radical (Fig. 4A). This highly reactive radical intermediate allows these enzymes to carry out various difficult chemical reactions, often to functionalize inactivated C-H bonds. The cofactor is coordinated by three cysteines in a relatively well conserved Cys-X_3_-Cys-X_2_-Cys motif and a SAM molecule (Fig. 1D).^20^

In addition to their [4Fe-4S] catalytic cluster, these SAM enzymes can also contain an auxiliary Fe-S cluster (either a [2Fe-2S] or a [4Fe-4S] cluster) that can serve as sulfur donor since free sulfur is toxic to the cell,^41^ or might have other functions including reducing the Fe-S cluster bound to the radical SAM, accepting electron during catalysis or coordinating the substrate molecule.^20,39^ The following part of this section focuses on sacrificial clusters used as a source of sulfur.

The biotin synthases that catalyze the conversion of dethiobiotin to biotin possess generally a [2Fe-2S] sacrificial auxiliary cluster (although a [4Fe-5S] cluster containing a ligated sulfide that is proposed to be used for biotin formation has been recently identified in biotin synthase of all obligate anaerobic organisms^105^) that is the source of sulfur atoms required for biotin formation.^19^ This synthesis starts with reductive cleavage of a SAM molecule, which is ligated to the [4Fe-4S] cluster, in L-methionine and 5ʹ-dA• (Fig. 4A). The radical then abstracts a hydrogen from dethiobiotin, generating a dethiobiotin C9 carbon radical that is quenched after being covalently attached to one of the two inorganic sulfur atoms of the [2Fe-2S] cluster, reducing one of the iron atoms in the process. A second SAM molecule then binds to the [4Fe-4S] cluster and is reductively cleaved. The generated 5ʹ-dA• abstracts a hydrogen from the C6 carbon of the 9-mercaptodethiobiotin intermediate leading to the production of a radical that attacks the sulfur atom attached to the C9 carbon, thus generating the thiophane ring of the biotin cofactor (Fig. 4B).^106–108^ In this reaction, the [2Fe-2S] is destroyed. The auxiliary cluster is coordinated by three cysteines and one arginine, which is likely critical in cluster retention or repair.^19,109^

Another example of SAM enzymes that possess an auxiliary cluster involved in sulfur donation include the bacterial lipoyl synthase LipA (ortholog of the human LIAS) that catalyzes the insertion of two sulfur atoms into the C6 and C8 positions of the octanoyl moiety bound to the lipoyl domains of lipoate-dependent enzymes. The auxiliary cluster of LipA is coordinated by three cysteines and one serine.^110^ Like the arginine residue in the biotin synthases, this type of coordination might facilitate the destabilization of the auxiliary cluster (and potentially its repair) and the transfer of the sulfur to the substrate.

## Challenges faced in the *de novo* identification of Fe-S proteins and new methods to identify Fe-S proteins in proteomes

### Hurdles in Fe-S protein identification

#### Diversity in cluster coordination

Fe-S clusters are generally coordinated to the protein by the side chain of cysteine residues but, occasionally, the coordination can involve the side chain of for instance a histidine, aspartic acid, glutamic acid, serine, or arginine or even of another ligand such as a SAM or substrate molecule as illustrated in Fig. 1. Members of each class of Fe-S proteins as seen in the Section “Biological functions of the [2Fe-2S] and [4Fe-4S] clusters” can present a common Fe-S coordination motif (e.g. the [4Fe-4S] cluster of the radical SAM enzymes is usually bound by a CX_3_CX_2_C motif) but between classes, ligation pattern can vary significantly. As a result, the identification of new Fe-S proteins, based in particular on primary sequences, remains a challenge especially when not fully coordinated by cysteine residues. Moreover, as Fe-S proteins, proteins with zinc cofactors also use cysteine and histidine residues as metal binding ligands, in often similar sequence motifs, making it difficult to predict whether the protein harbors a zinc or an Fe-S cofactor.^111^

#### Issues occurring during the production and purification of recombinant Fe-S proteins

Purification of Fe-S proteins is another hurdle in the identification of new Fe-S proteins. First, Fe-S clusters are generally oxygen sensitive and can be degraded during the purification. Often, purification typically relies on the overexpression of the protein in a heterologous expression system (e.g. *E. coli*). Overexpression of the proteins can affect Fe-S cluster loading and use of a heterologous expression system can also lead to mismetallation due to the possible absence of the native Fe-S cluster assembly machineries. Introducing these machineries in the host could be a solution to overcome this issue; however, they are complex machineries involving numerous factors and certain aspects of the Fe-S protein biogenesis remain unclear.^7,9^ Consequently, several Fe-S proteins have been expressed and purified from *E. coli* with zinc in their cluster binding sites (e.g. DNA polymerases and the scaffold protein ISCU^112–115^).

### Methods to identify new Fe-S proteins

#### Combination of spectroscopic, biochemical, and biophysical approaches

Once the protein of interest has been purified (preferentially under anaerobic conditions to prevent the degradation of the cluster by oxygen), a range of approaches can be employed to characterize the protein and its cofactor. Sometimes, when occupation rate by the cluster is low, chemical cluster reconstitution can be done prior further analysis to increase Fe-S cluster content. Spectroscopic and analytical techniques such as iron and sulfide content determination determined spectrophotometrically, inductively coupled plasma mass spectrometry (ICP–MS) and inductively coupled plasma optical emission spectrometry (ICP–OES), UV–Vis absorption spectroscopy, circular dichroism spectroscopy, EPR spectroscopy, Mössbauer spectroscopy, and native electrospray ionization mass spectrometry (nMS), can provide information on metal and ligand identity, stoichiometry, and oxidation state of the cluster.^111,116,117^ nMS has gained a lot of attention in the last years as it requires lesser amount of samples as compared to EPR and Mössbauer spectroscopies. Samples also do not require to be isotopically enriched, and this method can detect any oxidation state, and can simultaneously resolve all Fe-S cluster species in the samples, which is particularly useful when studying Fe-S sensors.^118^ NMR spectroscopy is another technique that can be employed to characterize Fe-S proteins, and contrary to EPR and Mössbauer, this technique provides dynamic structural information. However, the identification of NMR signals from residues near the Fe-S cluster is generally impaired in standard NMR experiments due to enhanced paramagnetic relaxation of their nuclei caused by the presence of the paramagnetic cofactor. To overcome this problem, several approaches have been developed including the combined use of standard and tailored ^1^H and ^13^C experiments that allow to reduce the blind sphere around the cluster.^119,120^ These techniques are often coupled with mutagenesis to allow the identification of coordinating ligands, and functional assays to verify that the metal binding the protein is the correct cofactor.

As already mentioned, numerous proteins have been mis-annotated. Examples include mitoNEET (Section “Repair proteins: the NEET protein mitoNEET and DNA glycosylases”) and nsp12, a subunit of the RNA-dependent RNA polymerase of SARS-CoV-2, that were thought to harbor zinc cofactors,^31^ and ATE1, involved in protein arginylation required for the degradation of proteins *via* the ubiquitin pathway, that was initially annotated as a heme binding protein.^33^ The mixture of spectroscopic, biochemical, and biophysical approaches enabled the correct annotation of these important proteins. Nsp12 was predicted to contain two zinc domains based on its primary sequence and modeling studies of a SARS-CoV-2 nsp12 homolog. Cryo-EM structures of nsp12 also modeled two zinc binding sites.^121–124^ However, the presence of two Lys-Tyr-Arg (LYR)-like motifs in nsp12 suggested the potential presence of an Fe-S cluster in the viral protein. Previous studies have indeed identified LYR-like motifs in the Fe-S protein SDHB (subunit of the respiratory complex II). This motif is thought to guide the Fe-S cluster transfer machinery to the apo-protein allowing Fe-S cluster insertion in human cells.^125–127^ Nsp12 and the component of Fe-S cluster transfer machinery HSC20 exhibit a strong binding interaction that is abrogated when the LYR tripeptide is replaced by three alanine suggesting that nsp12 could accept an Fe-S cluster.^31^ Of importance, it is worth keeping in mind that the LYR motif is not considered a universal trafficking motif. The protein nsp12 was then expressed in mammalian cells grown in the presence of ^55^Fe and a significant incorporation of ^55^Fe in the protein was observed; in opposite, this was significantly reduced in the 3xAla version of the protein.^31^ The optical spectrum of the purified nsp12 exhibited a peak at 420 nm characteristics of the presence of a [4Fe-4S] cluster that was confirmed by subsequent Mössbauer spectroscopy.^31^ This work showed that nsp12, which was considered as a zinc protein, binds in reality two [4Fe-4S] clusters. The discovery of an Fe-S cluster in ATE1 was unexpected but purification of the protein and subsequent spectroscopic analyses revealed the presence of an Fe-S cofactor that is oxygen-sensitive, displaying rapid decomposition from [4Fe-4S] to [2Fe-2S] cluster upon exposure to oxygen, and that is coordinated by four cysteines that are important for both cluster binding and arginylation activity.^33^

#### Proteome interrogation

Most of the studies (like the ones presented in Section “Combination of spectroscopic, biochemical, and biophysical approaches”) focus on the characterization of specific proteins. To accelerate the discovery of Fe-S proteins and thus provide a more complete overview of the cellular functions of this family of proteins, new approaches have been developed in the recent years to interrogate proteomes.

##### Isotopic tandem orthogonal proteolysis-activity-based protein profiling (isoTOP-ABPP)

Cysteines that are oxidized by ROS^128^ or are engaged in zinc^129^ or Fe-S cluster^34^ coordination lose their ability to react with an iodoacetamide-alkyne (IA-alkyne) probe. Assembly of Fe-S proteins is tightly regulated by cellular iron levels and required several factors for cluster synthesis and delivery to recipient apo-proteins. Therefore, by growing *E. coli* in standard vs. iron limited conditions, or by using wild-type bacteria vs. bacteria with impaired Isc pathway, and quantitatively assessing protein abundance and cysteine reactivity to the IA-alkyne probe in proteins from these cells (=isoTOP-ABPP strategy), Bak and Weerapana were able to monitor net changes in cysteine reactivity between the proteomes of the two biological systems (i.e. standard vs. iron depletion or impaired Isc machinery) providing deeper insight into the Fe-S proteins and their Fe-S biogenesis pathway.^34^ The isoTOP-ABPP strategy enabled the identification of previously unannotated Fe-S proteins as illustrated by TrhP (tRNA hydroxylase involved in the generation of hydroxyuridin) and DppD (part of the ABC transporter DppABCDF involved in dipeptide transport). These two proteins displayed a net reactivity increase in at least two of the six generated datasets with a greater than threefold net increase in reactivity in at least one. Increase in cysteine reactivity to the IA-alkyne probe is expected in Fe-S proteins from cells depleted in iron or Isc factors as there should be an increase in the level of apo-forms in these conditions, as compared to standard conditions. These proteins also contain at least three Cys residues (required for most of Fe-S cluster coordination); three of the five Cys residues present in TrhP displayed net increases in reactivity and were closely located in the 3D AlphaFold model of the protein, suggesting the possible presence of a cluster.^34^ UV–Vis absorption spectroscopy, EPR spectroscopy and ICP–OES analysis of purified proteins were used to confirm the presence of an Fe-S cluster in these proteins and validate the isoTOP-ABPP approach. TrhP expressed and purified from *E. coli* produced a brown solution typical of iron-containing proteins. ICP–OES analysis on the protein revealed the presence of four to five bound irons per monomer. The presence of a redox-active [4Fe-4S] cofactor was then confirmed by UV–Vis absorption and EPR spectroscopies of the oxidized (as-isolated) and dithionite-reduced proteins.^34^ So far, nearly 150 proteins in *E. coli* have been annotated as Fe-S proteins, which represent ∼3% of the proteome. The isoTOP-ABPP approach recovered ∼70% of known Fe-S proteins. The rest evaded detection possibly due to their low abundance, the lack of an ionizable IA-labeled peptide, or deeply buried cysteine ligands that are difficult for the IA-alkyne probe to access. These problems might be alleviated in the future by additional fractionation steps and more sensitive mass spectrometry instrumentation.

##### Computational methods with a focus on the Deep Mind's AlphaFold2 program

Computational methods are unaffected by the issues associated to the isoTOP-ABPP strategy and could be used as a complement. The deposition of numerous experimentally determined protein structures in Protein Data Bank has permitted the development of computational methods including Deep Mind's AlphaFold2 program.^130^ The AlphaFold2 software has revolutionized structural biology by providing 3D protein structure predictions for the whole proteome of 21 organisms, but cofactors were not included in these predictions. Recent works have analysed AlphaFold 3D models to know whether this program could accurately predict Fe-S cluster binding sites.^35,36^ In the study of Wehrspan *et al.*, the 362 311 protein structure predictions have been exploited to identify thousands of potential new binding sites for Fe-S (and zinc) cofactors.^35^ Using current knowledge on Fe-S clusters, a list of six variants of Fe-S cluster binding sites (i.e. “4Fe-4S Cys_4_” and “4Fe-4S Cys_3_” from PDB ID 3A38, “3Fe-4S Cys_3_” from 1WUI, “2Fe-2S Cys_4_” from 1N62, “2Fe-2S Cys_2_His_2_” from 3D89, and “2Fe-2S Cys_3_Asp_1_” from 1NEK) were exhaustively placed at all plausible locations within the different predicted protein structures.^35^ A similar approach has been previously employed to identify metal binding sites in crystallographic structures.^131^ The binding sites identified within 3D AlphaFold2 models were often assigned to one specific ligand (i.e. [2Fe-2S] cluster or [4Fe-4S] cluster), and as expected, based on current annotation in UniProt, there was a larger number of [4Fe-4S] than [2Fe-2S] cluster binding sites.^35^ Interestingly, an important number of [4Fe-4S] cluster binding sites identified in the prokaryotic proteomes appear to be coordinated by three Cys residues; the fourth iron could bind a water molecule as seen with the aconitases, a SAM molecule or another residue.^35^ Most known Fe-S proteins were recovered by the AlphaFold2 program. Indeed, 74% of known [4Fe-4S] clusters, coordinated by either three or four Cys residues in UniProt, were correctly identified in the AlphaFold2 structures. The non-recovery of the 26% known [4Fe-4S] cluster binding sites remaining could be explained notably by (i) “near misses” that could be identified by loosening the threshold, (ii) different ligands involved in the coordination of the same cluster between UniProt and AlphaFold2(according to the authors, the cluster coordination predicted by AlphaFold2 is sufficiently plausible that the current Uniprot annotation could be called into question), and (iii) the building of erroneous disulfide bonds between ligating Cys residues that should be relatively easy to identify.^35^ Regarding the [2Fe-2S] cofactors, 67% of annotated [2Fe-2S] clusters were recalled; the majority of the missed calls were due to the construction of erroneous disulfide bonds between the Cys residues coordinating the cluster.^35^ This seems to happen more frequently with [2Fe-2S] cluster binding sites than with [4Fe-4S] binding sites possibly because the two Cys residues involved are closer in the [2Fe-2S] cluster binding sites (∼3.5 Å) than in [4Fe-4S] cluster binding sites (∼6.3 Å). Of note, structures of proteins exceeding 2700 residues have not been reported except for the human proteome for which large proteins have been modeled using overlapping fragments of maximum 1400 residues that can be used to predict Fe-S cluster binding sites.^35^ Comparison with previous bioinformatics predictions^132,133^ showed strong overlaps with AlphaFold2-predicted Fe-S cluster binding sites for *E. coli*, and Cys residues predicted in the study to be involved in Fe-S cluster coordination presented generally a low reactivity to the IA-alkyne probe.^34,35^ A limitation, that also applies to the approaches mentioned in this section, is that coordinating residues might be shared between two monomers as seen for RsrR and NsrR (Section “Regulators of gene expression: the members of the Rrf2 family and the FNR as examples”) or even two different proteins as observed in the human mitoribosome.^16,17,134,135^ However, advances are being made to model multimeric protein complexes that could be further used to predict Fe-S cluster binding sites at the interface of two proteins.^136,137^ Computational predictions based on AlphaFold2 3D models can clearly contribute to the discovery of novel Fe-S proteins. These predictions might indeed help to prioritize candidate proteins identified in proteome-scale experiments such as in^34^ for further characterization. Actually, TrhP that was identified in^34^ as harboring a [4Fe-4S] cluster, is also predicted by AlphaFold2 to contain a [4Fe-4S] cluster.^35^ The predictions can also provide candidate proteins for further analysis. AlphaFold2 predicted that the human methyltransferase-like protein METTL17, a protein involved in the assembly of the mitoribosome, and the yeast orthologue Rsm22 harbor a [4Fe-4S] cluster. Recent study demonstrated that both proteins coordinate a [4Fe-4S] cluster.^138^ Numerous proteins have been predicted by AlphaFold as Fe-S clusters proteins but require now experimental confirmation.

## Conclusions

The Fe-S clusters are key cofactors in proteins in all kingdoms of life performing critical functions like electron transport, catalysis, sulfur donation and sensing, and can be important to maintain the protein structural integrity. Identification of Fe-S proteins is often overlooked due to the oxygen-sensitivity of their clusters and unusual coordination motifs, which make their prediction particularly challenging when based on the primary amino acid sequence. Another layer of complexity lies on the fact that the coordination of an Fe-S cluster can be shared between two proteins, making *in silico* prediction and experimental determination even more difficult. Examples include proteins of the mitoribosome and the Rrf2 family transcriptional regulators RsrR and NsrR.^16,17,134,135^ Diversity in cluster coordination patterns including in members of the same protein family (e.g. Rrf2 family) may be related to different functions of the clusters, even though this does not appear as a strict rule per se. For instance, clusters that enable coupling of electron transport and proton transfer have at least a histidine coordinating the cluster (e.g. Rieske centers, mitoNEET) while clusters that donate a sulfur (e.g. arginine for BioB), or participate in cluster transfer (e.g. histidine for mitoNEET) are coordinated by a residue conferring a certain level of cluster lability. In the case of Fe-S cluster-containing regulators, the nature of the signaling molecules might determine the residues involved in cofactor coordination. The aspartic acid ligand in NsrR is critical to sense NO and the absence of a fourth ligand in RirA is critical to enable cluster disassembly in low-iron condition.^16,69,71^ These coordination patterns might help create hypotheses regarding specific functions of the Fe-S proteins. Advances in the development of methods to predict Fe-S cluster binding sites *in silico* and *in cellulo* have contributed and will continue to contribute—with notably the development of tools to predict the structures of proteins exceeding 2700 residues and of multimeric protein complexes—to the identification of new Fe-S members, helping to better understand the roles of these important proteins.

## Data Availability

No new data were generated or analysed in support of this research.
